# Comparison of 3 Aging Metrics in Dual Declines to Capture All-Cause Dementia and Mortality Risk: Cohort Study

**DOI:** 10.2196/66104

**Published:** 2025-01-30

**Authors:** Anying Bai, Shan He, Yu Jiang, Weihao Xu, Zhanyi Lin

**Affiliations:** 1 School of Population Medicine and Public Health, Chinese Academy of Medical Sciences and Peking Union Medical College, Beijing, China; 2 Haikou Cadre's Sanitarium of Hainan Military Region, Haikou, China; 3 School of Health Policy and Management, Chinese Academy of Medical Sciences and Peking Union Medical College, Beijing, China; 4 School of Population Medicine and Public Health, Peking Union Medical College, Beijing, China; 5 Department of Geriatrics, Guangdong Provincial Geriatrics Institute, Guangdong Provincial People's Hospital, Guangdong Academy of Medical Sciences, Southern Medical University, No. 106, Zhongshan 2nd Road, Yuexiu District, Guangzhou, China, 0898-66571684

**Keywords:** gerontology, geriatrics, older adults, older people, aging, motoric cognitive risk syndrome, MCR, physio-cognitive decline syndrome, PCDS, cognitive frailty, CF, frailty, discrimination, risk factors, prediction, dementia risk, mortality risk

## Abstract

**Background:**

The utility of aging metrics that incorporate cognitive and physical function is not fully understood.

**Objective:**

We aim to compare the predictive capacities of 3 distinct aging metrics—motoric cognitive risk syndrome (MCR), physio-cognitive decline syndrome (PCDS), and cognitive frailty (CF)—for incident dementia and all-cause mortality among community-dwelling older adults.

**Methods:**

We used longitudinal data from waves 10-15 of the Health and Retirement Study. Cox proportional hazards regression analysis was employed to evaluate the effects of MCR, PCDS, and CF on incident all-cause dementia and mortality, controlling for socioeconomic and lifestyle factors, as well as medical comorbidities. Discrimination analysis was conducted to assess and compare the predictive accuracy of the 3 aging metrics.

**Results:**

A total of 2367 older individuals aged 65 years and older, with no baseline prevalence of dementia or disability, were ultimately included. The prevalence rates of MCR, PCDS, and CF were 5.4%, 6.3%, and 1.3%, respectively. Over a decade-long follow-up period, 341 cases of dementia and 573 deaths were recorded. All 3 metrics were predictive of incident all-cause dementia and mortality when adjusting for multiple confounders, with variations in the strength of their associations (incident dementia: MCR odds ratio [OR] 1.90, 95% CI 1.30‐2.78; CF 5.06, 95% CI 2.87‐8.92; PCDS 3.35, 95% CI 2.44‐4.58; mortality: MCR 1.60, 95% CI 1.17‐2.19; CF 3.26, 95% CI 1.99‐5.33; and PCDS 1.58, 95% CI 1.17‐2.13). The C-index indicated that PCDS and MCR had the highest discriminatory accuracy for all-cause dementia and mortality, respectively.

**Conclusions:**

Despite the inherent differences among the aging metrics that integrate cognitive and physical functions, they consistently identified risks of dementia and mortality. This underscores the importance of implementing targeted preventive strategies and intervention programs based on these metrics to enhance the overall quality of life and reduce premature deaths in aging populations.

## Introduction

The aging process encompasses various physiological declines across multiple systems. As the global population ages, the prevalence of dementia is rising, expected to reach 131 million individuals by 2050. This increase poses a significant economic challenge, potentially equating to 1.1% of the global gross domestic product by 2030 [1]. The lack of curative treatments for dementia and its substantial public health impact underscore the necessity for early detection to mitigate or delay its onset. Given dementia’s heterogeneous and lengthy preclinical phase, early screening and diagnosis in at-risk individuals are vital for disease management and caregiver preparedness [2].

Cognitive and physical declines with age often occur simultaneously, suggesting shared underlying mechanisms [3]. Researchers have developed metrics across molecular, phenotypic, and functional areas [4] to reflect the complex nature of aging accurately. Cognitive deterioration typically precedes dementia by several years, with evidence indicating that motor decline, especially in walking speed, can precede cognitive decline by over a decade [5,6]. Thus, composite aging metrics, encompassing both physical and cognitive functions, offer a promising method to gauge the functional status of the aging population. A meta-analysis highlighted an increased dementia risk in individuals with both physical frailty and cognitive impairment compared to those with cognitive impairment alone [7]. Therefore, composite aging metrics may serve as focal points for interventions aimed at preventing or delaying disability onset and enhancing the healthy lifespan of the elderly [8,9].

An ideal dementia screening tool for primary care should be brief, easily administered, acceptable to older individuals, and exhibit high sensitivity and specificity. Research on aging metrics that incorporate both cognitive and physical functions is gaining traction in gerontological studies due to their strong predictive power for adverse health outcomes. Various metrics have been introduced, such as cognitive frailty (CF) [10], motoric cognitive risk syndrome (MCR) [11], and physio-cognitive decline syndrome (PCDS) [12]. Despite their conceptual similarities, detailed assessments of their definitions and attributes are scarce, hindering their application in research and clinical settings.

Research has shown an increased incidence of concurrent gait and cognitive impairments in older adults susceptible to dementia [5]; however, the specific clinical traits of those experiencing both declines are not well-defined, and direct comparisons between different aging metrics have not been made. Additionally, the effectiveness of these metrics in identifying at-risk individuals and assessing the risk of adverse health events within the same population is not well understood. This lack of knowledge is clinically important for effectively categorizing older adults and identifying potentially reversible conditions in individuals with concurrent declines, thereby informing targeted strategies to slow dementia progression or reduce mortality rates. Consequently, our study seeks to fill these voids by comparing the risk of future dementia and all-cause mortality across 3 aging metrics and examining the predictive abilities of MCR, PCDS, and CF for adverse events among community-dwelling older adults without dementia at baseline, employing data from a broad population-based cohort.

## Methods

### Sample

This study analyzes data from waves 10‐15 of the Health and Retirement Study (HRS), the most extensive longitudinal study examining the aging experiences of Americans aged 51 and older. The HRS uses multi-stage probability sampling of U.S. households to obtain a nationally representative sample of adults in this age group [13]. It collects self-reported data on demographics, chronic health conditions, daily activities, disability status, and other health determinants at baseline and every 2 years thereafter. In 2006, the HRS began conducting enhanced face-to-face interviews that included physical performance assessments, biomarker collections, and a leave-behind questionnaire on psychosocial topics. Half of the households were chosen randomly for the enhanced interview in 2006, with the remainder selected in 2008, a process that continues in subsequent waves. Further information on the HRS’s recruitment strategies and design is detailed in previous literature [13].

**Figure 1.**
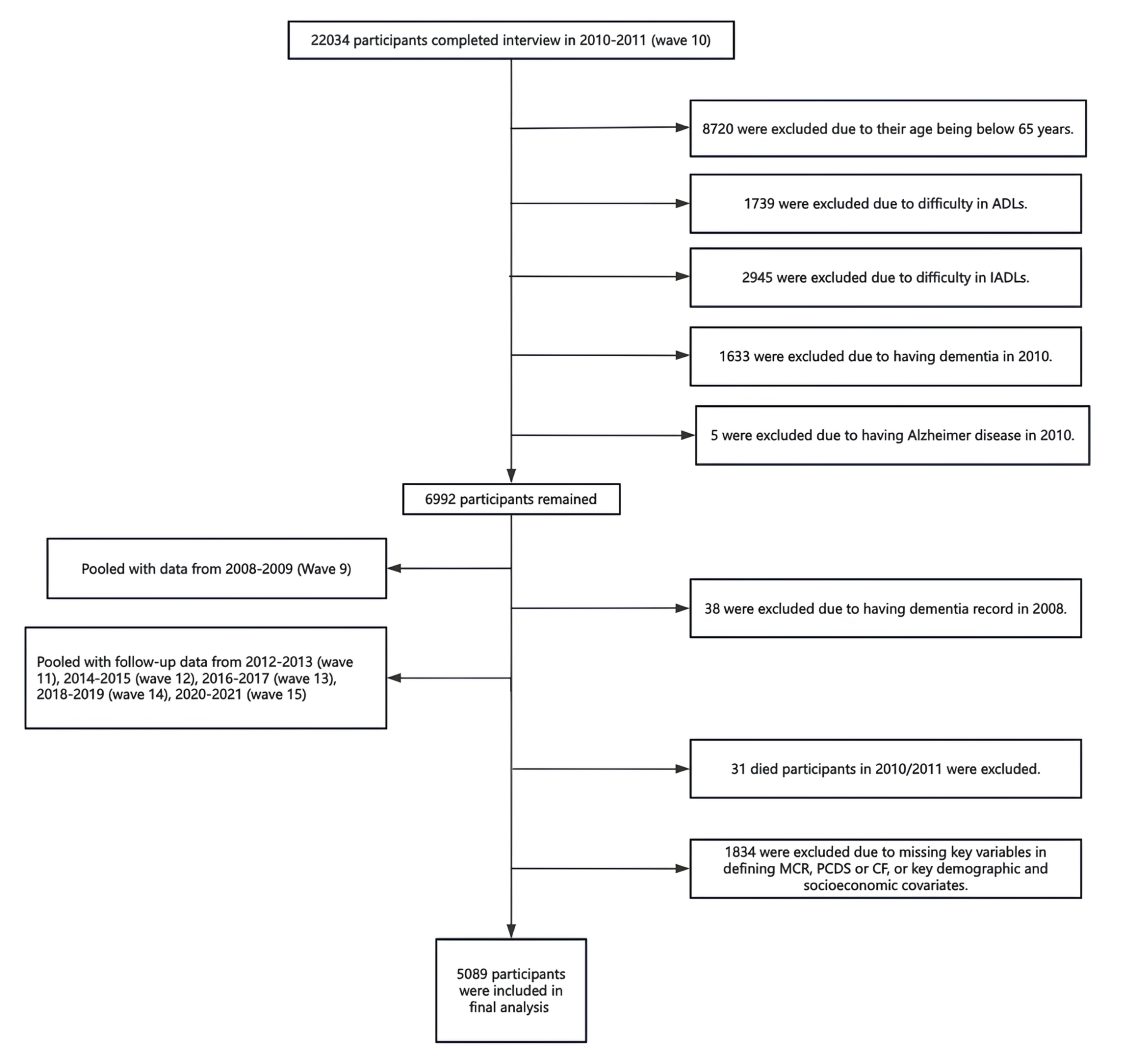
Study flowchart of participant selection. ADL: activity of daily living; IADL: instrumental activity of daily living; CF: cognitive frailty; MCR: motoric cognitive risk syndrome; PCDS: physio-cognitive decline syndrome.

The baseline for this analysis combined data from the 2008‐2009 (wave 9) and 2010‐2011 (wave 10) waves, marking the initial occasion respondents were asked about diagnoses of Alzheimer disease or dementia, instead of a “memory-related disease.” Mortality data has been available since 2011. A total of 22,034 individuals completed wave 10 and were followed biennially through to 2020‐2021 (wave 15). This study is a secondary analysis of the de-identified HRS public data, and the original HRS was approved by the University of Michigan Institutional Review Board. All participants signed the informed consent at the time of participation. Our final sample consisted of 2372 individuals who (1) were 65 years or older, (2) had complete baseline data on MCR, PCDS, and CF measures, (3) reported no difficulty with any activities of daily living and instrumental activities of daily living at baseline, (4) did not have Alzheimer disease or dementia at baseline, and (5) were alive in 2010 and 2011. Figure 1 shows the participant flow through each selection stage according to the inclusion criteria.

### Measures

#### Cognitive Function

Biennial cognitive function tests were administered by trained HRS interviewers either in-person or via telephone using the Modified Telephone Interview for Cognitive Status (TICS-m), a global cognition test modeled on the Mini-Mental State Examination. The TICS-m comprises immediate and delayed 10-noun free recall tests (score range: 0‐10 for each), a serial 7 subtraction test (score range: 0‐5), and a test of counting backward from 20 (score range: 0‐2). Higher scores denote better cognitive performance. During each assessment, HRS participants were categorized into normal cognition, mild cognitive impairment (MCI), or dementia based on established thresholds and comprehensive evaluations, including expert clinician adjudication from the Aging, Demographics, and Memory Study, a dementia sub-study within the HRS framework. Cognitive status was categorized into 3 distinct groups based on continuous scores [14], where scores from 12 to 27 indicated no impairment; scores between 7 and 11 signified cognitive impairment without dementia or MCI; and scores from 0 to 6 suggested dementia [15].

#### Motoric Cognitive Risk Syndrome

MCR was defined by subjective cognitive complaints coupled with slow gait in older adults who did not have a mobility disability or dementia [16]. In the HRS, gait speed (measured in meters per second) was determined from the time taken (in seconds) to walk a 2.5-meter course at a normal pace within participants’ homes. A slow gait was defined as performance ≥1 SD below the mean for the participant’s age and sex, a criterion previously used in HRS to identify MCR [17].

Subjective cognitive complaints were assessed using 2 questions: 1. “How would you rate your memory at the present time? Would you say it is excellent, very good, good, fair, or poor?” and 2. “Compared with the previous interview, would you say your memory is better now, about the same, or worse now than it was then?” Responses of ‘fair’ or ‘poor’ to the first question, or ‘worse’ to the second, were considered indicative of cognitive complaints.

#### Cognitive Frailty

CF was defined as the co-occurrence of physical frailty and MCI [18]. The concept of frailty was identified using the 5 criteria outlined by Fried et al [19] in the Cardiovascular Health Study: unintended weight loss, physical inactivity, exhaustion, weakness, and reduced speed. Unintended weight loss is recognized as either a 10% or greater reduction in BMI since the last measurement in 2008 or a current BMI less than 18.5 kg/m^2^. Physical activity levels were quantified by averaging the frequencies of activities at 3 levels of intensity—mild, moderate, and vigorous—weighted by the average metabolic equivalent of task (MET) scores for each intensity level: mild (1‐3 MET), moderate (4‐6 MET), and vigorous (7‐10 MET). Participants were deemed physically inactive if their average physical activity fell within the lowest 20%. Exhaustion was determined based on responses to 2 questions from the Center for Epidemiologic Studies Depression scale [20], including, “I could not get going” and “I felt that everything I did was an effort.” Muscle strength was assessed through the average of 2 grip strength measurements using a dynamometer on the dominant hand. Weakness was determined by grip strength falling below thresholds adjusted for BMI and gender, as established in the CHS. Reduced speed or slowness was defined as a speed <0.762 m/s for women taller than 159 cm or men taller than 173 cm and as <0.653 m/s for women 159 cm tall or less or men 173 cm tall or less, measured on a 2.5-m course [19,21]. Participants were considered to have missing data for physical measures if they were unable to perform the assessments due to the absence of suitable facilities or equipment or recent surgery. The diagnosis of frailty was based on the number of these criteria fulfilled, with those meeting 3-5 criteria classified as frail.

#### Physio-Cognitive Decline Syndrome

PCDS was defined as slowness or weakness (using cutoffs from the 2019 consensus update by the Asian Working Group for Sarcopenia), accompanied by cognitive performance that is at least 1.5 standard deviations below the mean for age-, sex-, and education-matched controls across all cognitive domains [12,22]. This assessment is based on comprehensive objective neuropsychological tests.

#### All-Cause Dementia and Mortality

The diagnosis of dementia was based on physician-diagnosed dementia and TICS scores ranging from 0 to 6. Mortality data were recorded, including the year and month of death, obtained from an exit interview or a spouse or partner’s core interview.

### Covariates

Covariates included sociodemographic factors, clinical characteristics, and health-related lifestyle behaviors, all of which were assessed at baseline. Sociodemographic characteristics included age (in years), sex (male or female), educational background (primary school or below, high school or equivalent, college and above), and marital status (married vs unmarried). Health-related lifestyle behaviors included excessive drinking, defined as more than 14 drinks per week for men and more than 7 drinks per week for women. Alcohol consumption was calculated by multiplying the number of days per week that alcohol (drink liquor or beer or wine or rice) was consumed by the number of drinks (liang or bottles or mugs) per day. Clinical characteristics included history of hypertension, diabetes, and heart disease. Hypertension was defined as systolic blood pressure ≥140 mm Hg, diastolic blood pressure ≥90 mm Hg, physical diagnosis, or antihypertensive medication use [23,24]. Diabetes was defined as having a diabetes diagnosis by a physician, being on treatment for diabetes, and having a fasting glucose level greater than or equal to 126 mg/dL and HbA_1c_ >6.5%. The presence of heart disease was determined via a physician’s diagnosis obtained through an in-person visit with study personnel via a questionnaire.

### Statistical Analysis

The incidence rates of all-cause dementia and mortality were calculated as the number of incident cases divided by the number of person-years of follow-up within the observation year (from 2008 to 2021). Differences between the MCR and non-MCR groups, PCDS and non-PCDS groups, and CF and non-CF groups were assessed using a 2-sided, independent *t*-test and the *χ*^2^ test. To evaluate the impact of MCR, PCDS, and CF on the occurrence of all-cause dementia and mortality, we employed Cox proportional hazards regression analysis. The observation period extended from the index date to the earliest of the following: onset of dementia, death, or the conclusion of the observation period (December 31, 2018). Adjusted hazard ratios (AHRs) for MCR, PCDS, and CF in predicting the onset of dementia and all-cause mortality were calculated, accounting for covariates in an initially unadjusted model. Subsequent adjustments for covariates were made in 2 stages: Model 1 adjusted for age and gender; Model 2 further incorporated socioeconomic (education level and marital status), lifestyle (excessive drinking), and medical conditions (hypertension, diabetes, heart disease, and stroke). To assess and compare the predictive accuracy of all models, discrimination, defined by the model’s ability to differentiate between individuals who develop dementia and those who do not, was quantified using Harrell C-statistic, taking survival into account.

Several sensitivity analyses were conducted to verify the stability of our findings. First, to focus on new cases and reduce reverse causation bias, individuals diagnosed with dementia or who died within 2 years of follow-up (sensitivity analysis I) were excluded. Second, to address the competing risk of death for dementia occurrences, Fine and Gray competing risk models were used [25], comparing these to the results from Cox proportional hazards regression models (sensitivity analysis II). The sub-distribution hazard function, defined at time *t*, represents the immediate risk of event *k* among individuals not previously experiencing event *k*. Third, to minimize selection bias, associations within individual samples with complete data on MCR, PCDS, and CF were analyzed separately (sensitivity analysis III). Statistical analyses were performed using 2-tailed tests with a significance level of *P*<.05 and 95% CIs, employing Stata (version 17) for all statistical procedures.

### Ethical Considerations

This investigation has been conducted in accordance with the ethical standards of the Declaration of Helsinki, as well as national and international guidelines. The study has been approved by the Institutional Review Board at the University of Michigan (approval number: HUM00061128). All participants were provided with detailed information about the study, including its purpose, procedures, potential risks and benefits, and their rights to withdraw at any time. Written informed consent was obtained from all participants prior to their involvement in the study. To ensure privacy and confidentiality, all data collected were anonymized and deidentified. No identifying information was retained or published. Protective measures were in place to safeguard participant information, including secure storage of data and restricted access to study records. Participants were not compensated for their involvement in this study. The research was conducted on a voluntary basis, and no financial or other incentives were provided.

## Results

### Baseline Characteristics

The study participants’ baseline characteristics are outlined in Table 1. The initial cohort comprised 2367 individuals, featuring prevalence rates for MCR, PCDS, and CF at 5.4% (n=121), 6.3% (n=140), and 1.3% (n=31), respectively. Among these, CF patients were the oldest on average (75.7, SD 6.1 years), with the distribution of men being 52.89% (64/121) in the MCR group, 47.14% (66/140) in the PCDS group, and 58.06% (10/31) in the CF group. During the follow-up, 573 (24.2%) patients died. The proportions of all-cause dementia for MCR, PCDS, and CF were 24.8% (30/121), 33.6% (47/140), and 41.9% (13/31), respectively.

**Table 1.** Characteristics of included patients at baseline according to 3 aging metrics.

Variable	Non-MCR^a^ (n=2246)	MCR (n=121)	*P* value	Non-PCDS^b^ (n=2227)	PCDS (n=140)	*P* value	Non-CF^c^ (n=2336)	CF (n=31)	*P* value
Age (years), mean (SD)	73.78 (5.65)	73.29 (5.54)	.17	73.72 (5.62)	74.29 (6.02)	.08	73.73 (5.63)	75.65 (6.11)	.52
Male, n (%)	1086 (48.35)	64 (52.89)	.33	1084 (48.68）	66 (47.14)	.73	1132 (48.46)	18 (58.06)	.29
Educational background, n (%)			.001			.80			<.001
Illiterate	286 (12.73)	27 (22.31)		297 (13.34）	16 (11.43)		301 (12.89）	12 (38.71)	
Primary or above	1286 (57.26)	73 (60.33)		1276 (57.30%)	83 (59.29)		1343 (57.49)	16 (51.61)	
Secondary or above	674 (30.01)	21 (17.36)		654 (29.37)	41 (29.29)		692 (29.62)	3 (9.68)	
Medical history, n (%)									
Hypertension	1398 (62.24)	91 (75.21)	.004	1392 (62.51)	97 (69.29)	.11	1466 (62.76)	23 (74.19)	.19
Diabetes	431 (19.19)	25 (20.66)	.69	410 (18.41)	46 (32.86)	<.001	446 (19.09)	10 (32.26)	.07
Heart disease	605 (26.94)	47 (38.84)	.004	615 (27.62)	37 (26.43)	.76	638 (27.31)	14 (45.16)	.03
Excessive drink	1436 (63.94)	95 (78.51)	.001	1428 (64.12)	103 (73.57)	.02	1509 (64.60)	22 (70.97)	.46
Incident all-cause dementia	311 (13.85)	30 (24.79)	.001	294 (13.20)	47 (33.57)	<.001	328 (14.04)	13 (41.94)	<.001
Mortality	530 (23.60)	43 (35.54)	.003	526 (23.62)	47 (33.57)	.008	556 (23.80）	17 (54.84)	<.001

a MCR: motoric cognitive risk syndrome.

b PCDS: physio-cognitive decline syndrome.

c CF: cognitive frailty.

### Relationships of MCR, PCDS, and CF With Incident Dementia and All-Cause Mortality

Overall, there were 341 incident dementia cases during follow-up, for an overall incidence rate of 19.52 (95% CI 17.56-21.71) per 1000 person-years. There were 573 cases that died during follow-up, for an overall incidence rate of 30.14 (95% CI 27.77-32.71) per 1000 person-years (Table 2). The incidence rates of all-cause dementia among MCR, CF, and PCDS patients were 38.04, 108.11, and 55.80 per 1000 person-years, respectively—significantly higher than those observed in the relatively healthy control group. Similarly, the incidence rates of all-cause mortality were 47.99, 100.79, and 44.70 per 1000 person-years for MCR, CF, and PCDS patients, respectively—again, markedly higher than in the healthy controls. Table 2 also demonstrates significant associations of the 3 conditions with increased risks of incident dementia and all-cause mortality in various models (all *P* values <.001). CF had the highest AHR for both outcomes (5.06; 95% CI 2.87-8.92 for dementia, and 3.26; 95% CI 1.99‐5.33 for mortality). Participants with dual decline experienced a two to threefold increased risk of dementia progression (AHR: 1.90‐2.22 in the MCR group; 3.21‐3.35 in the PCDS group) compared with those without dual decline. The AHR for all-cause mortality ranged from 1.60 to 1.84 in the MCR group and from 1.56 to 1.63 in the PCDS group. The trend was consistent across models: AHRs for dementia and mortality increased from the unadjusted model to adjusted model 1, then decreased upon further adjustment for covariates, including socioeconomic status, lifestyle factors, and medical comorbidities. Sensitivity analyses I (Table S1 in Multimedia Appendix 1) and III (Table S2 in Multimedia Appendix 1) consistently showed an elevated risk of dementia and mortality for MCR, CF, and PCDS. However, in sensitivity analysis II, the standardized hazard ratios for MCR and CF were not statistically significant post-adjustment (Table S3 in Multimedia Appendix 1).

**Table 2.** Associations between 3 aging metrics, incident all-cause dementia, and all-cause mortality.

	Total sample	MCR^a^		CF^b^		PCDS^c^	
		No	Yes	No	Yes	No	Yes
All-cause dementia							
Events and sample size, n/N	341/2367	311/2246	30/121	328/2336	13/31	294/2227	47/140
Incidence (95% CI)^d^	19.52 (17.56‐21.71)	18.65 (16.69‐20.84)	38.04 (26.59‐54.40)	18.91 (16.97‐21.07)	108.11 (62.77‐186.18)	17.68 (15.77‐19.83)	55.80 (41.93‐74.27)
Unadjusted HR^e^ (95% CI)	—^f^	Ref.^g^	2.03 (1.40‐2.95)	Ref.	6.23 (3.57‐10.88)	Ref.	3.21 (2.36‐4.37)
Model 1^h^: adjusted HR (95% CI)	—	Ref.	2.22 (1.53‐3.24)	Ref.	6.62 (3.79‐11.57)	Ref.	3.29 (2.41‐4.48)
Model 2^i^: adjusted HR (95% CI)	—	Ref.	1.90 (1.30‐2.78)	Ref.	5.06 (2.87‐8.92)	Ref.	3.35 (2.44‐4.58)
All-cause mortality							
Events and sample size, n/N	573/2367	530/2246	43/121	556/2336	17/31	526/2227	47/140
Incidence (95% CI)	30.14 (27.77‐32.71)	29.26 (26.87‐31.86)	47.99 (35.59‐64.71)	29.51 (27.16‐32.07)	100.79 (62.66‐162.13)	29.29 (26.89‐31.90)	44.70 (33.58‐59.49)
Unadjusted HR (95% CI)	—	Ref.	1.68 (1.23‐2.29)	Ref.	3.93 (2.42‐6.38)	Ref.	1.56 (1.16‐2.11)
Model 1: adjusted HR (95% CI)	—	Ref.	1.84 (1.35‐2.51)	Ref.	4.04 (2.49‐6.57)	Ref.	1.63 (1.21‐2.19)
Model 2: adjusted HR (95% CI)	—	Ref.	1.60 (1.17‐2.19)	Ref.	3.26 (1.99‐5.33)	Ref.	1.58 (1.17‐2.13)

a MCR: motoric cognitive risk syndrome.

b CF: cognitive frailty.

c PCDS: physio-cognitive decline syndrome.

d Incidence rates = events per 1000 person-years.

e HR: hazard ratio.

f Not applicable.

g Ref: reference.

h Model 1 adjusted for age and gender.

i Model 2 further adjusted for educational background, marital status, excessive drinking, hypertension, diabetes, and heart disease.

### Discriminations of MCR, PCDS, and CF for All Outcomes

The discrimination abilities of MCR, PCDS, and CF regarding all-cause dementia and mortality are detailed in Table 3. The C-index for PCDS in identifying all-cause dementia was 0.732 (95% CI 0.703-0.760), outperforming the C-indices for MCR and CF (PCDS vs MCR: 0.012, 95% CI −0.001 to 0.025; *P*=.08; CF vs MCR: 0.005, 95% CI −0.004 to 0.013; *P*=.35). In contrast, MCR’s C-index for identifying all-cause mortality was the highest among the 3, at 0.727 (95% CI 0.706-0.748). Similar to the case with incident dementia, the differences in discrimination ability among MCR, PCDS, and CF for all-cause mortality were not statistically significant.

**Table 3.** Harrell C-index for Cox regression models predicting incident all-cause dementia, and all-cause mortality.

	C-index^a^	95% CI	*P* value	Difference	*P* value
Incident all-cause dementia					
MCR^b^	0.7194	0.6915 to 0.7472	<.001	Reference	
PCDS^c^	0.7315	0.7028 to 0.7602	<.001	0.0121 (−0.0009 to 0.0252)	.08
CF^d^	0.7239	0.6959 to 0.7520	<.001	0.0046 (−0.0038 to 0.0129)	.35
All-cause mortality					
MCR	0.727	0.7061 to 0.7479	<.001	Reference	
PCDS	0.7259	0.7048 to 0.7469	<.001	−0.0012 (−0.0054 to 0.0030)	.59
CF	0.7254	0.7044 to 0.7465	<.001	−0.0016 (−0.0054 to 0.0022)	.41

a All indexes were estimated in models adjusted for age, gender, educational background, marital status, excessive drinking, hypertension, diabetes, and heart disease

b MCR: motoric cognitive risk syndrome.

c PCDS: physio-cognitive decline syndrome.

d CF: cognitive frailty.

**Figure 2.**
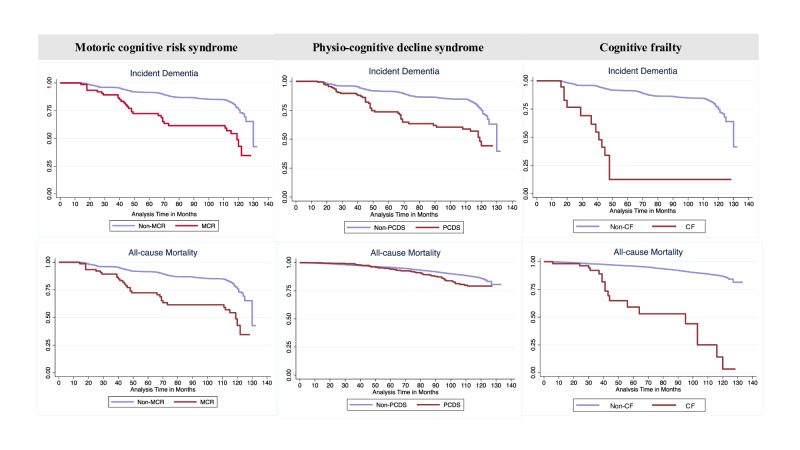
Kaplan-Meier survival curve depicting the proportion of participants remaining dementia-free or surviving during follow-up, comparing motoric cognitive risk syndrome (MCR), physio-cognitive decline syndrome (PCDS), and cognitive frailty (CF) patients with their respective healthy control groups.

The Kaplan-Meier curve, depicted in Figure 2, shows the duration to incident dementia or all-cause mortality, stratified by MCR, PCDS, or CF, and healthy controls, with adjustments made for all covariates. Both curves demonstrate a decline over the follow-up period, with a notably pronounced decrease observed among patients (all *P* values for log rank <.001).

## Discussion

### Principal Findings

Analyzing data from a large-scale cohort in the United States, this study is the first to illustrate that MCR, PCDS, and CF have significant associations with incident dementia and all-cause mortality within the same population, despite the inherent heterogeneity of these metrics. Among them, CF emerged as the highest-risk group for both dementia and mortality, with markedly elevated incidence rates and the highest AHRs (5.06 for dementia and 3.26 for mortality), identifying CF as the most vulnerable subgroup. These findings offer valuable insights into the future health trajectories of frail individuals with cognitive impairments and highlight the need for targeted interventions and early health education to mitigate dementia and mortality risks in later life at lower costs. While PCDS showed the strongest predictive ability for dementia and MCR for mortality, the differences in predictive accuracy were not statistically significant. Implementing appropriate management strategies can help alleviate the health care burden for individuals with varying cognitive and physical conditions.

To forestall unhealthy aging, health care systems need to identify individuals at risk of functional declines that are still preventable or reversible. Our results are in line with prior studies, indicating that MCR syndrome is linked with a 90% increased risk of incident dementia and a 60% higher risk of mortality, even after adjustments for demographics, SES, and cardiovascular comorbidities (hypertension, diabetes, and heart disease). Consistent with research from Chung et al, the prevalence of hypertension and heart disease was significantly higher in the MCR group, highlighting the importance of regular screening to manage cardiovascular risk factors and delay dementia onset [26,27]. These observations further emphasize the potential neurodegenerative nature of MCR pathologic changes [28].

CF has been associated with an increased risk of dementia and mortality, as demonstrated by a meta-analysis revealing that older adults with CF face higher mortality and dementia risks than their healthier counterparts [29]. However, the absence of a standardized assessment tool for CF hampers its use in widespread epidemiological studies. Using the frailty phenotype alongside the MMSE, although common, proves to be time-intensive and presents challenges for deployment in community and hospital settings [30]. Furthermore, the lower prevalence of CF compared to other measures (1.31% for CF vs 5.91% for PCDS vs 5.11% for MCR) in the community-dwelling older adult cohort indicates difficulties in identifying a substantial number of at-risk individuals for interventions in similar groups.

In contrast to CF, PCDS, with its precise definition encompassing physical decline and cognitive impairment, provides a more targeted approach. By focusing on the mobility aspects of frailty linked to worse cognitive outcomes and increased mortality risk, researchers can more accurately identify the target population, concentrating on specific causal mechanisms [31,32]. PCDS serves as an advantageous focus for multidomain interventions, integrating physical activity, cognitive training, nutritional counseling, and disease management education to improve the condition of vulnerable but potentially reversible older individuals [33]. A recent Singapore study that used a 12-week dual-task exercise program to examine the potential reversibility of CF, MCR, and PCDS also found clinical improvements in PCDS, but the longer-term effects remain uncertain [34]. Nonetheless, Lee et al [35] demonstrated that PCDS was related to a 6-year, but not 3-year, incidence of dementia, even when using inverse probability weighting analysis to account for bias from missing data. This finding implies a latency period for the development of dementia, highlighting this interval as potentially crucial for preventive interventions.

While PCDS demonstrated the best predictive performance for incident dementia, MCR emerged as a superior predictor for all-cause mortality. The choice between these 2 metrics may depend on practical considerations, with MCR offering several unique advantages for clinical use. MCR is simpler and more efficient to assess, unaffected by education levels or learning effects, which enhances its credibility and reliability as a screening tool. In contrast, the comprehensive cognitive assessments required for PCDS diagnosis can be labor-intensive and require specific skills, which may limit their use in community or primary care settings. Given MCR’s higher predictive accuracy for all-cause mortality and its ease of implementation, it is well-suited for use in routine health care assessments, particularly in primary health care settings. Additionally, research suggests that while handgrip strength declines earlier in aging, walking speed in later life is a stronger predictor of mortality [36]. This further supports MCR’s higher predictive accuracy for all-cause mortality.

Despite representing a prodromal phenotype of accelerated aging, MCR remains an important target for intervention to prevent poor outcomes in older adults. MCR offers incremental validity in predicting dementia beyond what is provided by MCI subtypes [16] and individual components such as subjective cognitive complaints or slow gait [16,37]. Our findings suggest that MCR could serve as a simpler, more efficient tool to identify individuals at higher risk for dementia, especially given its independence from educational background or learning effects. The motor components of MCR, such as the time-up-and-go test or one-leg-standing test, are reliable and valid methods for quantifying motor function, making MCR an easily implementable screening tool in various health care settings. However, one study has shown that the MCR subtype defined by the 5-times-sit-to-stand test, which also includes a balance component, was less effective in predicting cognitive decline compared to MCR defined by slow gait [38]. Further research is needed to determine how different gait and balance assessments influence the identification of individuals at risk for dementia, and whether integrating MCR into routine geriatric assessments could enhance early intervention strategies in aging populations.

Early and appropriate intervention might postpone the onset of dementia or reduce its risk, particularly given the lengthy incubation period associated with the development of dementia. Our results could aid clinicians in the early implementation of screening and prevention strategies and inform government decisions on community health prevention. The strength of this study lies in its extensive, well-delineated cohort, featuring longitudinal assessments, uniform measures, and verified outcomes. To our knowledge, this is the first study to assess the predictive accuracy of MCR, PCDS, and CF regarding dementia incidence and all-cause mortality within the same cohort. The reliability and consistency of our findings, supported by sensitivity analyses, bolster the study’s validity. Nonetheless, several limitations should be acknowledged. First, the study lacked objective neuropsychological testing [39] and did not account for some potential confounding factors, such as *APOE* genotype or imaging biomarkers [40,41]. However, our sensitivity analysis—excluding participants diagnosed with dementia or those who died within 2 years of the index date—strengthened the robustness of our conclusions. Second, while we used data from a large longitudinal cohort, the final sample size of 2372 individuals may limit the representativeness of our findings. Additionally, the prevalence rates of MCR (5.4%), PCDS (6.3%), and CF (1.3%) in our sample may be higher than those in the general older population, introducing potential selection bias. To address this, sensitivity analyses were conducted within individual subgroups with complete data on MCR, PCDS, and CF. These analyses included larger sample sizes and showed lower prevalence rates for the 3 conditions, aligning more closely with those observed in the general population. The robustness of these results further confirmed our conclusions, enhancing their validity and generalizability while mitigating potential selection bias. Future research could benefit from larger, more diverse, and representative samples to enhance generalizability. Third, the potential influence of mortality on dementia risk assessment must be considered. Individuals who died during follow-up could bias dementia outcome evaluations. To address this competing risk, we employed Fine and Gray competing risk models and compared the results with those from Cox proportional hazards regression models as part of our sensitivity analysis. This approach ensured that our findings remained robust and accounted for the impact of mortality on dementia risk associations. Finally, variations in syndrome definitions and differences in the prevalence of the 3 aging metrics within the population could affect their predictive power for adverse outcomes. This variability may limit the generalizability of our results to other populations. To mitigate this, sensitivity analyses within subgroups with complete data on MCR, PCDS, and CF were conducted, reinforcing the reliability of the associations between these aging metrics and adverse health outcomes.

### Conclusions

The integration of cognitive and physical functions into aging metrics consistently indicates risks for incident dementia and all-cause mortality, despite their significant differences, underscoring their utility for effective risk stratification in research and clinical settings. Patients with CF represent the most vulnerable subgroup, highlighting the need for prioritized preventive strategies and interventions. Meanwhile, MCR emerged as a particularly efficient and accurate screening tool for both dementia and mortality. These findings emphasize the importance of implementing targeted prevention and intervention programs based on these metrics to enhance quality of life and reduce premature mortality among aging populations. Further research on the longitudinal dynamics of these aging metrics in relation to dementia, mortality, and other outcomes is essential for a deeper understanding of their long-term impact.

## Supplementary material

10.2196/66104 Multimedia Appendix 1 Supplementary materials.

## Abbreviations

AHR: adjusted hazard ratio
CF: cognitive frailty
HRS: Health and Retirement Study
MCI: mild cognitive impairment
MCR: motoric cognitive risk syndrome
MET: metabolic equivalent of task
OR: odds ratio
PCDS: physio-cognitive decline syndrome
TICS-m: Modified Telephone Interview for Cognitive Status

## References

[1] Zeisel J, Bennett K, Fleming R (2020). World Alzheimer report 2020: design, dignity, dementia: dementia-related design and the built environment.

[2] Borson S, Frank L, Bayley PJ (2013). Improving dementia care: the role of screening and detection of cognitive impairment. Alzheimers Dement.

[3] Robertson DA, Savva GM, Kenny RA (2013). Frailty and cognitive impairment--a review of the evidence and causal mechanisms. Ageing Res Rev.

[4] Ferrucci L, Levine ME, Kuo PL, Simonsick EM (2018). Time and the metrics of aging. Circ Res.

[5] Montero-Odasso MM, Barnes B, Speechley M (2016). Disentangling cognitive-frailty: results from the gait and brain study. J Gerontol A Biol Sci Med Sci.

[6] Dumurgier J, Artaud F, Touraine C (2016). Gait speed and decline in gait speed as predictors of incident dementia. J Gerontol A Biol Sci Med Sci.

[7] Zheng L, Li G, Gao D (2020). Cognitive frailty as a predictor of dementia among older adults: a systematic review and meta-analysis. Arch Gerontol Geriatr.

[8] Cano A (2015). Cognitive frailty, a new target for healthy ageing. Maturitas.

[9] Ruan Q, Yu Z, Chen M, Bao Z, Li J, He W (2015). Cognitive frailty, a novel target for the prevention of elderly dependency. Ageing Res Rev.

[10] Kelaiditi E, Cesari M, Canevelli M (2013). Cognitive frailty: rational and definition from an (I.A.N.A./I.A.G.G.) International Consensus Group. J Nutr Health Aging.

[11] Verghese J, Annweiler C, Ayers E (2014). Motoric cognitive risk syndrome: multicountry prevalence and dementia risk. Neurology (ECronicon).

[12] Chung CP, Lee WJ, Peng LN (2021). Physio-cognitive decline syndrome as the phenotype and treatment target of unhealthy aging. J Nutr Health Aging.

[13] Heeringa SG, Connor JH (1995). Technical Description of the Health and Retirement Survey Sample Design.

[14] Langa KM, Plassman BL, Wallace RB (2005). The Aging, Demographics, and Memory Study: study design and methods. Neuroepidemiology.

[15] Crimmins EM, Kim JK, Langa KM, Weir DR (2011). Assessment of cognition using surveys and neuropsychological assessment: the Health and Retirement Study and the Aging, Demographics, and Memory Study. J Gerontol B Psychol Sci Soc Sci.

[16] Verghese J, Wang C, Lipton RB, Holtzer R (2013). Motoric cognitive risk syndrome and the risk of dementia. J Gerontol A Biol Sci Med Sci.

[17] Ayers E, Verghese J (2016). Motoric cognitive risk syndrome and risk of mortality in older adults. Alzheim Dementia.

[18] Gaspar PM, Campos-Magdaleno M, Pereiro AX, Facal D, Juncos-Rabadán O (2022). Cognitive reserve and mental health in cognitive frailty phenotypes: Insights from a study with a Portuguese sample. Front Psychol.

[19] Fried LP, Tangen CM, Walston J (2001). Frailty in older adults: evidence for a phenotype. J Gerontol A Biol Sci Med Sci.

[20] Radloff LS (1977). The CES-D Scale: a self-report depression scale for research in the general population. Appl Psychol Meas.

[21] Bandeen-Roche K, Xue QL, Ferrucci L (2006). Phenotype of frailty: characterization in the women’s health and aging studies. J Gerontol A Biol Sci Med Sci.

[22] Chen LK, Arai H (2020). Physio-cognitive decline as the accelerated aging phenotype. Arch Gerontol Geriatr.

[23] Chen S, Sudharsanan N, Huang F, Liu Y, Geldsetzer P, Bärnighausen T (2019). Impact of community based screening for hypertension on blood pressure after two years: regression discontinuity analysis in a national cohort of older adults in China. Br Med J.

[24] Geldsetzer P, Manne-Goehler J, Marcus ME (2019). The state of hypertension care in 44 low-income and middle-income countries: a cross-sectional study of nationally representative individual-level data from 1·1 million adults. Lancet.

[25] Fine JP, Gray RJ (1999). A proportional hazards model for the subdistribution of a competing risk. J Am Stat Assoc.

[26] Ngandu T, Lehtisalo J, Solomon A (2015). A 2 year multidomain intervention of diet, exercise, cognitive training, and vascular risk monitoring versus control to prevent cognitive decline in at-risk elderly people (FINGER): a randomised controlled trial. The Lancet.

[27] Crous-Bou M, Minguillón C, Gramunt N, Molinuevo JL (2017). Alzheimer’s disease prevention: from risk factors to early intervention. Alz Res Therapy.

[28] Sekhon H, Allali G, Launay CP (2019). Motoric cognitive risk syndrome, incident cognitive impairment and morphological brain abnormalities: systematic review and meta-analysis. Maturitas.

[29] Bu Z, Huang A, Xue M, Li Q, Bai Y, Xu G (2021). Cognitive frailty as a predictor of adverse outcomes among older adults: a systematic review and meta-analysis. Brain Behav.

[30] Zhang XM, Wu XJ, Cao J, Jiao J, Chen W (2022). Association between cognitive frailty and adverse outcomes among older adults: a meta-analysis. J Nutr Health Aging.

[31] Wu YH, Liu LK, Chen WT (2015). Cognitive function in individuals with physical frailty but without dementia or cognitive complaints: results from the I-Lan Longitudinal Aging Study. J Am Med Dir Assoc.

[32] Liu LK, Chen CH, Lee WJ (2018). Cognitive frailty and its association with all-cause mortality among community-dwelling older adults in Taiwan: results from I-Lan Longitudinal Aging Study. Rejuvenation Res.

[33] Chen LK, Hwang AC, Lee WJ (2020). Efficacy of multidomain interventions to improve physical frailty, depression and cognition: data from cluster-randomized controlled trials. J Cachexia Sarcopenia Muscle.

[34] Merchant RA, Chan YH, Hui RJY (2021). Motoric cognitive risk syndrome, physio-cognitive decline syndrome, cognitive frailty and reversibility with dual-task exercise. Exp Gerontol.

[35] Lee WJ, Peng LN, Lin MH (2022). Six-year transition of physio-cognitive decline syndrome: results from I-Lan Longitudinal Aging Study. Arch Gerontol Geriatr.

[36] Zaccardi F, Franks PW, Dudbridge F, Davies MJ, Khunti K, Yates T (2021). Mortality risk comparing walking pace to handgrip strength and a healthy lifestyle: a UK Biobank study. Eur J Prev Cardiol.

[37] Verghese J, Lipton RB, Hall CB, Kuslansky G, Katz MJ, Buschke H (2002). Abnormality of gait as a predictor of non-Alzheimer’s dementia. N Engl J Med.

[38] Sekhon H, Launay CP, Chabot J, Allali G, Beauchet O (2018). Motoric cognitive risk syndrome: could it be defined through increased five-times-sit-to-stand test time, rather than slow walking speed?. Front Aging Neurosci.

[39] Alexander LK, Lopes B, Ricchetti-Masterson K, Yeatts KB (2015). Sources of systematic error or bias: Information bias. ERIC Notebook.

[40] Baumgart M, Snyder HM, Carrillo MC, Fazio S, Kim H, Johns H (2015). Summary of the evidence on modifiable risk factors for cognitive decline and dementia: a population-based perspective. Alzheimers Dement.

[41] Campbell NL, Unverzagt F, LaMantia MA, Khan BA, Boustani MA (2013). Risk factors for the progression of mild cognitive impairment to dementia. Clin Geriatr Med.

[42] HRS data portal. Health and Retirement Study.

