# Supplementary material for: Comparison of 3 Aging Metrics in Dual Declines to Capture All-Cause Dementia and Mortality Risk: Cohort Study
Source: JMIR Aging. 2025 Jan 30;8:e66104. doi: 10.2196/66104 (PMC11801771; doi:10.2196/66104)
Supplement: Multimedia Appendix 1 [file aging-v8-e66104-s001.docx]

**Supplement Materials**

**A comparison of three aging metrics in dual declines to capture all-cause dementia and mortality risk**

**Table S1. Associations between three aging metrics, incident dementia and all-cause mortality after excluding the participants developing dementia or deceased in the first two years.**

**Table S2. Associations between three aging metrics, incident dementia and all-cause mortality in individual sample.**

**Table S3. Competing risk regression model (all-cause mortality was set as the competing risk for dementia).**

**Table S1.** Associations between three aging metrics, incident dementia and all-cause mortality after excluding the participants developing dementia or deceased in the first two years.

|  | **Unadjusted HR^a^ (95%CI)** | **Model 1: adjusted HR (95%CI)** | **Model 2: adjusted HR (95%CI)** |
| --- | --- | --- | --- |
| **Incident All-cause Dementia** | |  |  |
| Motoric Cognitive Risk Syndrome |  |  |  |
| No | Ref. | Ref. | Ref. |
| Yes | 2.03 (1.31 - 3.14) | 2.27 (1.46 - 3.52) | 2.04 (1.31 - 3.19) |
| Cognitive Frailty |  |  |  |
| No | Ref. | Ref. | Ref. |
| Yes | 6.56 (3.24 - 13.30) | 7.38 (3.63 - 14.99) | 6.73 (3.29 - 13.74) |
| Physio-Cognitive Decline Syndrome | |  |  |
| No | Ref. | Ref. | Ref. |
| Yes | 3.02 (2.09 - 4.38) | 3.29 (2.27 - 4.77) | 3.40 (2.33 - 4.97) |
| **All-cause Mortality** |  |  |  |
| Motoric Cognitive Risk Syndrome |  |  |  |
| No | Ref. | Ref. | Ref. |
| Yes | 1.83 (1.33 - 2.50) | 2.01 (1.47 - 2.76) | 1.76 (1.28 - 2.42) |
| Cognitive Frailty |  |  |  |
| No | Ref. | Ref. | Ref. |
| Yes | 4.35 (2.64 - 7.17) | 4.63 (2.80 - 7.63) | 3.76 (2.26 - 6.25) |
| Physio-Cognitive Decline Syndrome | |  |  |
| No | Ref. | Ref. | Ref. |
| Yes | 1.70 (1.26 - 2.30) | 1.80 (1.33 - 2.44) | 1.73 (1.27 - 2.35) |

1. HR: Hazard Ratios.

**Table S2.** Associations between three aging metrics, incident dementia and all-cause mortality in individual sample.

|  | **Proportion of Non-missing Values (%)^a^** | **Total No. of Participants** | **No. of Events** | **Unadjusted HR^b^ (95%CI)** | **Model 1^c^: adjusted HR (95%CI)** | **Model 2^d^: adjusted HR (95%CI)** |
| --- | --- | --- | --- | --- | --- | --- |
| **All-cause Dementia** |  |  |  |  |  |  |
| MCR | 74.42% | 5152 | 864 |  |  |  |
| No |  |  |  | Ref. | Ref. | Ref. |
| Yes |  |  |  | 1.87  (1.48 - 2.37) | 1.92  (1.51 - 2.43) | 1.53  (1.21 - 1.95) |
| Cognitive Frailty | 40.59% | 2810 | 448 |  |  |  |
| No |  |  |  | Ref. | Ref. | Ref. |
| Yes |  |  |  | 3.81  (3.10 - 4.67） | 3.25  (2.63 - 4.00） | 2.67  (2.16 - 3.30） |
| Physio-cognitive decline syndrome | 90.68% | 6278 | 1163 |  |  |  |
| No |  |  |  | Ref. | Ref. | Ref. |
| Yes |  |  |  | 2.67  (2.19 - 3.27) | 2.87  (2.35 - 3.51) | 3.05  (2.49 - 3.73) |
| **All-cause Mortality** |  |  |  |  |  |  |
| MCR | 74.42% | 5152 | 1549 |  |  |  |
| No |  |  |  | Ref. | Ref. | Ref. |
| Yes |  |  |  | 1.68  (1.40 - 2.01) | 1.67  (1.39 - 2.00) | 1.47  (1.22 - 1.76) |
| Cognitive Frailty | 40.59% | 2810 | 739 |  |  |  |
| No |  |  |  | Ref. | Ref. | Ref. |
| Yes |  |  |  | 2.28  (1.92 - 2.71） | 1.79  (1.50 - 2.14） | 1.62  (1.36 - 1.93） |
| Physio-cognitive decline syndrome | 90.68% | 6278 | 2201 |  |  |  |
| No |  |  |  | Ref. | Ref. | Ref. |
| Yes |  |  |  | 1.42  (1.19 - 1.71) | 1.46  (1.22 - 1.75) | 1.46   - 1. - 1.75) |

1. Proportions of non-missing values were calculated as population 65 years or older, reported no difficulty with any activities of daily living (ADLs) and instrumental activities of daily living (IADLs) at baseline, did not have AD or dementia at baseline, and alive in 2010/2011 (N = 6923, see Figure 1 for more details) divided by total number of participants in each individual sample.
2. Model 1 adjusted for age and gender.
3. HR: Hazard Ratios.
4. Model 2 further adjusted for educational background, marital status, excessive drinking, hypertension, diabetes, and heart disease.

**Table S3.** Competing risk regression model (all-cause mortality was set as the competing risk for dementia).

|  | **Unadjusted HR (95%CI)** | **Model 1^a^: adjusted SHR^b^ (95%CI)** | **Model 2^c^: adjusted SHR (95%CI)** | |
| --- | --- | --- | --- | --- |
| Motoric Cognitive Risk Syndrome |  |  |  |  |
| No | Ref. | Ref. | Ref. |  |
| Yes | 1.58 (1.01 - 2.48) | 1.65 (1.06 - 2.59) | 1.50 (0.95 - 2.38) | |
| Cognitive Frailty | |  |  |  |
| No | Ref. | Ref. | Ref. |  |
| Yes | 2.37 (1.04 - 5.37) | 2.17 (0.94 - 5.04) | 1.72 (0.72 - 4.13) | |
| Physio-cognitive decline syndrome | | |  |  |
| No | Ref. | Ref. | Ref. |  |
| Yes | 2.34 (1.59 - 3.41) | 2.26 (1.53 - 3.33) | - 1. 1.52 - 3.31) | |

1. Model 1 adjusted for age and gender.
2. SHR, sub-distribution hazard ratio.
3. Model 2 further adjusted for educational background, marital status, excessive drinking, hypertension, diabetes, and heart disease
